# Arterial Hypertension and Diabetes Mellitus in COVID-19 Patients: What Is Known by Gender Differences?

**DOI:** 10.3390/jcm10163740

**Published:** 2021-08-23

**Authors:** Tiziana Ciarambino, Filippina Ciaburri, Venere Delli Paoli, Giuseppe Caruso, Mauro Giordano, Maria D’Avino

**Affiliations:** 1 Internal Medicine Department, Hospital of Marcianise, 81037 Caserta, Italy; 2 Hypertension Unit, Cardarelli Hospital, Naples 80110, Italy; filippinalibetta@alice.it (F.C.); giuseppe.caruso@gmail.com (G.C.); dott.mariadavino@gmail.com (M.D.); 3 Internal Medicine Department, Cardarelli Hospital, 80110 Naples, Italy; venere.dellipaoli@gmail.com; 4 Internal Medicine Department, University of Campania, L. Vanvitelli, 80110 Naples, Italy; mauro.giordano@unicampania.it

**Keywords:** COVID-19 infection, diabetes mellitus, arterial hypertension, SARS-CoV-2, outcome, comorbidity

## Abstract

Background. Severe acute respiratory syndrome coronavirus-2 (SARS-CoV-2) has infected >160 million people around the world. Hypertension (HT), chronic heart disease (CHD), and diabetes mellitus (DM) increase susceptibility to SARS-CoV-2 infection. Aims. We designed this retrospective study to assess the gender differences in hypertensive diabetic SARS-CoV-2 patients. We reported data, by gender differences, on the inflammatory status, on the hospital stays, intensive care unit (ICU) admission, Rx and CT report, and therapy. Methods. We enrolled 1014 patients with confirmed COVID-19 admitted into different Hospitals of Campania from 26 March to 30 June, 2020. All patients were allocated into two groups: diabetic-hypertensive group (DM-HT group) that includes 556 patients affected by diabetes mellitus and arterial hypertension and the non-diabetic- non-hypertensive group (non-DM, non-HT group) comprising 458 patients. The clinical outcomes (i.e., discharges, mortality, length of stay, therapy, and admission to intensive care) were monitored up to June 30, 2020. Results. We described, in the DM-HT group, higher proportion of cardiopathy ischemic (CHD) (47.5% vs. 14.8%, respectively; *p* < 0.0001) and lung diseases in females compared to male subjects (34.8% vs. 18.5%, respectively; *p* < 0.0001). In male subjects, we observed higher proportion of kidney diseases (CKD) (11% vs. 0.01%, respectively; *p* < 0.0001), a higher hospital stay compared to female subjects (22 days vs. 17 days, respectively, *p* < 0.0001), a higher admission in ICU (66.9% vs. 12.8%, respectively, *p* < 0.0001), and higher death rate (17.3% vs. 10.7%, respectively, *p* < 0.0001). Conclusion. These data confirm that male subjects, compared to female subjects, have a higher hospital stay, a higher admission to ICU, and higher death rate.

## 1. Introduction

Coronavirus disease 2019 (COVID-19) has infected over 160 million people around the world [1]. Hypertension (HT), chronic heart disease (CHD), and diabetes mellitus (DM), particularly in elderly people, increase susceptibility to severe COVID-19 [2]. In particular, about 12.8% to 31.2% of patients with COVID-19 had pre-existing hypertension as far as anamnestic chronic cardio-cerebrovascular disease (16.4%) and diabetes (9.7%) [3,4]. Because these patients seem to develop severe forms of COVID-19, an increased rate of mortality in this clinical setting has also been reported [5]. Several studies, in fact, described an increased risk of death in patients with DM and HT [6,7], and from a clinical point of view, inpatients with COVID-19 and comorbidities such as DM and HT, appear to have needed greater intensity of care [7,8]. Yet, not univocal data are present, concerning gender differences in inpatients with COVID-19 with DM and HT as comorbidities. A recent metanalysis [9] reported that males affected by COVID-19 are more likely to develop adverse symptoms or prognosis [9]; therefore, the authors suggest gender as a risk factor for prognosis of COVID-19 [9]. 

In this report, we designed a retrospective observational study to assess the gender differences in DM-HT SARS-CoV-2 inpatients in an Italian cohort of subjects observed in different Hospitals as dedicated units for COVID-19 in Southern Italy. We reported data on gender differences for clinical presentation and evolution (e.g., duration of hospitalization and/or admission in an intensive care unit (ICU) and/or discharge and/or therapeutic regimens) regarding inflammation (e.g., levels of C-reactive protein).

## 2. Methods

We enrolled 1014 patients with confirmed COVID-19 admitted into different Hospitals of Campania (different Campania Hospital as dedicated Intensive care unit (ICU) for COVID-19) from 26 March to 30 June, 2020. All patients were allocated into two groups: diabetic-hypertensive group (DM-HT group), which includes 556 patients affected by diabetes mellitus and arterial hypertension, and the nondiabetic-non-hypertensive group (non-DM, non-HT group), comprising 458 patients. Hypertensive patients (HT group) received a diagnosis of hypertension before admission to our hospitals, according to Guideline Hypertension ESC-ESH 2018, and all patients were treated with common antihypertensive therapy (such as ACE.I, calcium antagonist, etc). Diabetic patients received a diagnosis of diabetes before admission to our hospitals, according to ADA 2021. Not-diabetic, not-hypertensive patients (not-DM-HT group) were patients consecutively enrolled in the study population. We acquired informed consent from all patients. Baseline characteristics are shown in Table 1. We now report the definition of ischemic heart disease, such as that described by the “Fourth Universal Definition of Myocardial Infarction” [10]: the lung disease is defined as reported by the 2002 American Thoracic Society and European Respiratory Society consensus statement [11], while the definition and classification of chronic kidney disease (CKD) have evolved over time, and current international guidelines define this condition as decreased kidney function shown by glomerular filtration rate (GFR) of less than 60 mL/min per 1·73 m^2^ [12]. The definition of ischemic ictus is reported by [13].

The clinical outcomes (i.e., discharges, mortality, length of stay, CT and Rx report, and therapy, admission on the intensive care) were monitored up to June 30, 2020. During hospitalization, we extracted, from electronic medical records, data on the use of drugs, demographic data, medical history, exposure history, comorbidities, symptoms, signs, laboratory findings, and treatment measures (i.e., antiviral therapy, LMWH therapy, corticosteroid therapy). The status of disease followed the guideline of SARS-CoV-2, reported as follows [5]:

- Mild type (with slight clinical symptoms but no imaging findings of pneumonia);
- Common type (with fever, respiratory tract, and imaging findings of pneumonia);
- Severe type (with any of the following conditions):
  - Respiratory distress with respiratory frequency > 30 times/min;
  - Finger oxygen saturation at rest 93% AA; or
  - Oxygenation index (PaO_2_/FiO_2_) < 300 mmHg;
- Critical type (with any of the following conditions):
  c. Respiratory failure that requires mechanical ventilation;
  d. Shock and other organ failures that require intensive care unit.

Laboratory parameters, including complete blood count, hs-CRP (high-sensitivity C-reactive protein), arterial blood gas analysis (ABG), myocardial injury markers, coagulation profile, serum biochemical tests (including renal and liver function, lactate dehydrogenase), PCT (procalcitonin), and BNP (b-type natriuretic peptide), were measured according to the manufacturer’s instructions. Blood tests are repeated during hospitalization at admission, after 48h, and at discharge.

## 3. Statistical Analysis

Statistical significance α was fixed to 0.05. Numerical variables were described as mean (SD) and compared through unpaired t-test. We used the unpaired student’s *t*-test.

## 4. Results

**Figure 1:**
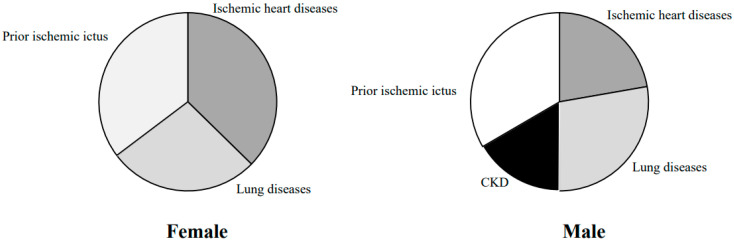
Comorbidity by gender differences.

The clinical characteristics of our population, by gender differences, are described in Table 1. In female DM-HT COVID-19 patients, compared to males, we observed a higher frequency of ischemic heart diseases, which aligns with another study [14] (47.5% versus 14.8%, respectively, *p*: 0.001). Additionally, we report a higher prevalence of lung diseases in DM-HT COVID-19 patients, compared to males (34.8% versus 18.5%, respectively, *p*: 0.001) and prior ischemic ictus (44.9% versus 22.2%, respectively, *p*: 0.001). Conversely, in male DM-HT COVID-19 patients, compared to females, we observed a higher proportion of renal diseases (0.1% versus 11.1%, respectively, *p*: 0.001) (Figure 1).

**Figure 2:**
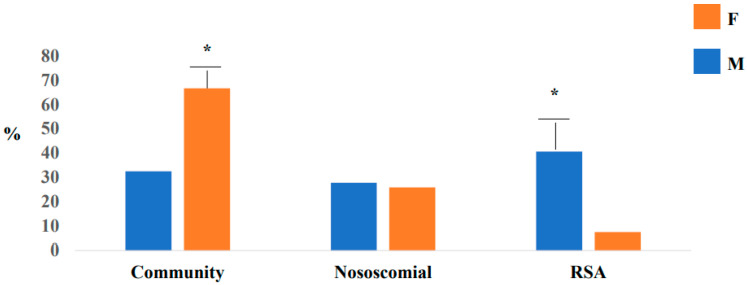
Epidemiological link by gender differences, * *p* < 0.001. RSA (Residence assistance for elderly).

In relation to symptoms, we reported that fever is a common symptom in female patients (85.2% vs. 42%, *p* < 0.0001, respectively) compared to male patients. Of note, that the hospital stay was higher in the male compared to the female group (22 days vs. 17 days; *p* < 0.0001). In relation to epidemiological link, we observed higher proportion on community infection in male subjects, compared to females (66.7% vs. 32.5%, *p* < 0.0001), and a higher proportion of RSA infection in male (40.7% vs. 7.4%, *p* < 0.0001) (Figure 2). We documented differences in relation to computerized tomography (CT) report by gender differences. In male subjects, compared to females, a higher proportion of atypical CT was reported (100% vs. 88.9%, respectively, *p* < 0.0001). In Table 2, we report the characteristics baseline in two groups (DM-HT and non-DM non -HT) COVID-19 patients.

### Outcomes

**Figure 3:**
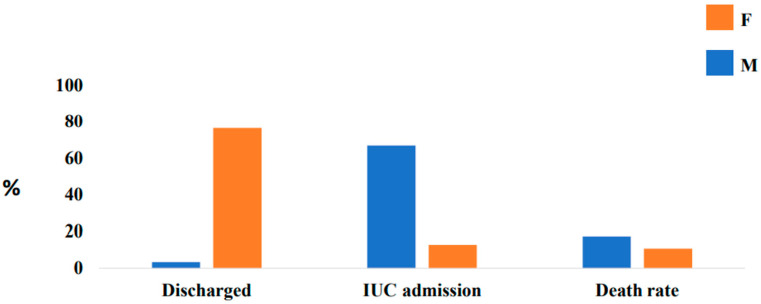
OUTCOME by gender differences.

Outcomes by gender differences in DM-HT groups, were reported in Table 3 and are described here. In particular, in females, we report that 18.3% were of critical, 21.5% were severe, and 62% were mild. However, in our study, we described that 76.5% of females, compared to 3.4% of males, were discharged (*p* < 0.0001); 66.9% of males, compared to 12.8% of females, were admitted into the intensive care unit (*p* < 0.0001), as reported in Figure 3. However, males have a higher death rate (17.3% vs. 10.7%, *p* < 0.0001). In male subjects, we observed a higher proportion on the O_2_ therapy (51.8% vs. 29.1%, *p* < 0.0001, respectively), and high-flow oxygen therapy with nasal cannula (100.0% vs. (93.7%, *p* < 0.0001, respectively) compared to female subjects. In relation to pharmacological therapy, we report a higher proportion on hydroxychloroquine in male subjects (96.3% vs. 44.9%, *p* < 0.0001) compared to female subjects. However, we observed a higher proportion of the Darunavir/ritonavir (7.4% vs. 0.0%, *p* < 0.0001, respectively) and on LMWH therapy (70.4% vs. 30.5%, *p* < 0.0001) in male subjects compared to female subjects.

## 5. Discussion

It has been reported that pre-existing morbidities increase the severity of COVID-19 infection [15]. In particular, pre-existing cardiovascular (CV) risk factors, such as diabetes mellitus and cardiopathy, seem to be crucial predictors for COVID-19 severity. The fatality rate, in fact, was much higher in COVID-19 patients with comorbidities [16]. It is not known if hypertension could be a risk factor for susceptibility to contract COVID-19, but it is more prevalent in severe COVID-19 cases and in subjects with a negative outcome [8]. In this regard, different studies reported that hypertension and diabetes represent a crucial comorbidity in COVID-19 patients [17,18,19,20,21,22]. The relationship between diabetes, hypertension, and COVID-19 infection seems to be related to the role of ACE2 [22,23], which may act as a regulator of hypertension and also a receptor for SARS-CoV-2. The angiotensin–renin system, in fact, is dysregulated in subjects with hypertension and SARS-CoV-2 infection [24]. It has been described that hypertension is associated with higher plasma interleukin-6 concentration in COVID-19 patients [25,26]. Levels of IL-6 and other cytokines are not only prominent in patients with severe COVID-19 disease [27,28] but could have profound CV consequences in patients with COVID-19 [29,30]. Mortality rate may increase by 10.5% for patients with CV risk factors, 7.3% for those with diabetes, and 6% for subjects with hypertension [31]. The histopathologic findings of diffuse alveolar damage (DAD) from COVID-19 appear to be indistinguishable from other causes of DAD. The acute/exudative phase of DAD is characterized by inflammatory cell-mediated alveolar damage with alveolar edema and/or hemorrhage, capillary congestion, and hyaline membranes with or without microvascular thrombi. The proliferative/organizing phase of DAD shows type II pneumocyte hyperplasia, reactive pneumocytes, alveolar wall thickening, and myofibroblast proliferation, whereas the chronic/fibrotic phase shows honeycomb lung with collagenous fibrosis of alveolar spaces and an interstitium with thickening of the alveolar wall along with squamous metaplasia of alveoli.

We designed this retrospective study to assess the gender differences in diabetic-hypertensive SARS-CoV-2 inpatients. In our population, we found in female DM-HT COVID-19 patients, compared to males, a higher proportion of ischemic heart diseases, lung diseases, and prior ischemic ictus. Conversely, in male DM-HT COVID-19 patients, compared to females, we observed a higher proportion of renal diseases. According to another study [29], male DM-HT COVID-19 patients had a higher hospital stay (22 days vs. 17 days; *p* < 0.001), a higher proportion of admission in intensive care units (ICUs) (66.9% vs. 12.8%, *p* < 0.001), and a higher death rate (17.2% vs. 10.7%, *p* < 0.001). Different studies reported that males seem to be more susceptible to COVID-19-related complications, and they represent between 50% and 82% of the hospitalized patients [9,22]. It is likely that our male patients were admitted more into ICUs because they are characterized by a higher proportion of atypical computerized tomography (CT), by higher proportion on the O_2_ therapy, and by high-flow oxygen therapy with nasal cannula. However, male subjects are characterized by a delay in access to care and they are admitted into hospitals in the advanced stages of the disease.

However, in relation to the epidemiological link, we observed, for the first time, a higher proportion of community infection in male subjects, compared to females, a higher proportion of RSA infection in smales. We report, for the first time, that male DM-HT subjects have a higher proportion of hydroxychloroquine and antiviral drugs compared to female subjects. However, our males needed aggressive therapies, probably in relation to their severity of disease and delay in access to care. These differences may depend on different crucial factors. In particular, we report that our male DM-HT subjects are characterized by a higher proportion of atypical tomography computerized (CT). These data could be related to probable delay in access to care for male patients; i.e., only in the advanced stages of the disease. In addition, even the atypical CT report, in males, could lead to the administration of a more aggressive therapy. Therefore, gender differences should be one of the criteria to select the appropriate therapies for the appropriate patients.

## 6. Conclusions and Study Limitations

The mechanisms by which hypertension and diabetes induce poor clinical outcomes in male COVID-19 patients remain obscure. We reported that DM-HT can be an important risk factor for progression and unfavorable outcomes in COVID-19 patients. The novelty of our paper is that we reported preliminary gender differences in relation to epidemiological link, CT report, and outcome (such as death rate, admission on the ICU, hospital stay). Of course, our study has several limitations. First, interpretation of our findings might be limited by the sample size and the retrospective observational nature of the study. Second, males received hydroxychloroquine more frequently, and it could be responsible for a worse clinical situation. Third, the influence of diabetes and hypertension on severity and outcome in COVID-19 patients, by gender differences, is not clear because of the large gap in the evidence. Finally, the follow-up period of this study is short to assess the prognosis of COVID-19. Large studies with comprehensive analysis of all risk factors and longer follow up are necessary to answer all questions that have been raised.

## Figures and Tables

**Table 1:** Characteristics baseline in DM-HT COVID-19 patients by gender differences. SBP (systolic blood pressure); DBP (diastolic blood pressure); DM (diabetes mellitus); CKD (chronic kidney disease); ICM (body mass index).

	Female (n. 270)	Male (n. 286)	*p* Value
Age (mean)	76.0 (56.0–86.0)	63.0 (51.5-70.0)	<0.0001
Ischemic heart disease	128 (47.5%)	42 (14.8%)	<0.0001
Lung Disease	93 (34.8%)	53 (18.5%)	<0.0001
CKD	0 (0.01%)	32 (11.1%)	<0.0001
Prior Ischemic ictus	121 (44.9%)	63 (22.2%)	<0.0001
Stay hospital (days)	17.0 (9.0, 23.0)	22.0 (16.0, 33.0)	<0.0001
SBP (mmHg)	135 (130–145)	130 (125–135)	<0.0001
DBP (mmHg)	78 (74–88)	72 (70–84)	<0.0001
Basal glicemia	135 (140–165)	130 (135–160)	<0.0001
ICM (kg/m^2^)	25 (24.5–25.5)	26 (25.5–26.5)	0.024
Smoking status (%)	40 (15)	45 (16)	<0.0001

**Table 2:** Characteristics baseline in two groups (DM-HT and non-DM non -HT) of COVID-19 patients. SBP (systolic blood pressure); DBP (diastolic blood pressure); ICM (body mass index).

	DM-HT Group (n. 556)	Non-DM Non-HT Group (n. 458)	*p* Value
Age (mean)	76.0 (56.0–86.0)	67 (51.0-83.0)	<0.0001
SBP (mmHg)	140 (130–145)	110 (105–115)	<0.0001
DBP (mmHg)	90 (85–100)	77 (70–84)	<0.0001
Basal glicemia	137 (140–165)	110 (105–115)	<0.0001
ICM (kg/m^2^)	27 (26.5–27.5)	26.0 (25.5–27.5)	0.024
Smoking status (%)	100 (18)	71 (16)	0.0039

**Table 3:** Gender differences in DM-HT groups. ICU (intensive care unit), CT (computerized tomography), high-flow nasal cannula (HFNC).

	Male (n. 286)	Female (n. 270)	*p* Value
*Epidemiological Link*			
Community	180 (66.7%)	87 (32.5%)	<0.0001
Nosocomial	79 (27.8%)	70 (25.9%)	0.0016
RSA	116 (40.7%)	20 (7.4%)	<0.0001
*Symptoms*			
Fever	120 (42.0%)	230 (85.2%)	<0.0001
Dyspnea	148 (51.9%)	40 (14.8%)	<0.0001
*Rx lung*			
Atypical	242 (84.8%)	69 (25.7%)	<0.0001
Typical	44 (15.2%)	200 (74.3%)	<0.0001
*CT lung*			
Atypical	286 (100.0%)	240 (88.9%)	<0.0001
Typical	0 (0.0%)	30 (11.1%)	<0.0001
PF ratio < 300 (%)	58 (20.3%)	40 (14.8%)	<0.0001
Discharge at home (%)	9 (3.14%)	206 (76.5%)	<0.0001
ICU admission (%)	191 (66.9%)	34 (12.8%)	<0.0001
Death rate (%)	49 (17.3%)	28 (10.7%)	<0.0001
O_2_ therapy	140 (51.8%)	83 (29.1%)	<0.0001
HFNC	270 (100.0%)	267 (93.7%)	<0.0001
Hydroxychloroquine	128 (44.9%)	260 (96.3%)	<0.0001
Darunavir/ritonavir	21 (7.4%)	0 (0.0%)	Ns
LMWH therapy	201 (70.4%)	82 (30.5%)	<0.0001

## Data Availability

Not applicable.
